# Design of an *In Vitro* Model to Screen the Chemical Reactivity Induced by Polyphenols and Vitamins during Digestion: An Application to Processed Meat

**DOI:** 10.3390/foods10092230

**Published:** 2021-09-20

**Authors:** Eléna Keuleyan, Aline Bonifacie, Philippe Gatellier, Claude Ferreira, Sylvie Blinet, Aurélie Promeyrat, Gilles Nassy, Véronique Santé-Lhoutellier, Laëtitia Théron

**Affiliations:** 1 Institut National de Recherche Pour l’Agriculture, l’Alimentation et l’Environnement (INRAE), UR370 Qualité des Produits Animaux, 63122 Saint Genès-Champanelle, France; elena.keuleyan@inrae.fr (E.K.); aline.bonifacie@inrae.fr (A.B.); philippe.gatellier@inrae.fr (P.G.); claude.ferreira@inrae.fr (C.F.); sylvie.blinet@inrae.fr (S.B.); veronique.sante-lhoutellier@inrae.fr (V.S.-L.); 2 IFIP—Institut du Porc, 7 Avenue du Général De Gaulle, 94700 Maisons Alfort, France; 3 IFIP—Institut du Porc, La Motte au Vicomte, BP 35104, 35561 Le Rheu, France; aurelie.promeyrat@ifip.asso.fr (A.P.); gilles.nassy@ifip.asso.fr (G.N.)

**Keywords:** N-nitrosation, lipid oxidation, processed meats, nitrite, differential spectrophotometry, emulsion, nitroso-compounds, polyphenols, vitamins, reformulation

## Abstract

Processed meats’ nutritional quality may be enhanced by bioactive vegetable molecules, by preventing the synthesis of nitrosamines from N-nitrosation, and harmful aldehydes from lipid oxidation, through their reformulation. Both reactions occur during digestion. The precise effect of these molecules during processed meats’ digestion must be deepened to wisely select the most efficient vegetable compounds. The aim of this study was to design an *in vitro* experimental method, allowing to foresee polyphenols and vitamins’ effects on the chemical reactivity linked to processed meats’ digestion. The method measured the modulation of end products formation (specific nitroso-tryptophan and thiobarbituric acid reactive substances (TBARS)), by differential UV-visible spectrophotometry, according to the presence or not of phenolic compounds (chlorogenic acid, rutin, naringin, naringenin) or vitamins (ascorbic acid and trolox). The reactional medium was supported by an oil in water emulsion mimicking the physico-chemical environment of the gastric compartment. The model was optimized to uphold the reactions in a stable and simplified model featuring processed meat composition. Rutin, chlorogenic acid, naringin, and naringenin significantly inhibited lipid oxidation. N-nitrosation was inhibited by the presence of lipids and ascorbate. This methodology paves the way for an accurate selection of molecules within the framework of processed meat products reformulation.

## 1. Introduction

Processed meat is a highly consumed meat product, and provides with very digestible proteins, oxidizable lipids, iron, and optional additives such as sodium nitrite. Yet, the latter is at the origin of consumers’ concerns as it is involved in several chemical reactions jeopardizing the nutritional quality of processed meats. The digestive tract displays an enhancing environment for those reactions, which may lead toward negative health patterns.

Particularly, N-nitrosation is the reaction between secondary amines (stemming from proteolysis) and nitrosonium ions (from sodium nitrite) leading to nitrosamines. Some of them have been identified as carcinogenic and the rest possibly for human health by the International Agency for Research on Cancer (2015) [1]. Yet, the main exposure to nitrosamines seems to correspond to an endogenous formation of the substances in the digestive tract [2]. By another hand, processed meat lipids undergo the oxidative chain reaction [3], which is also enhanced in the gastrointestinal tract and boosts the formation of aldehydes in the gastric compartment [4], some of which are cytotoxic and genotoxic [5,6]. Both of those reactions concern processed meats for their composition and formulations.

Nonetheless, increasingly more vegetable extracts aimed to be used in the meat industry emerge on the market and are used to supplement processed meats [7] in order to newly formulate those products. This trend is part of global movement whereby food products are reformulated with less processed ingredients, less food additives, and more naturalness [7,8]. Indeed, there is ample evidence that vegetable food products (such as tea, coffee, vegetables, or fruit juices), which are rich in active compounds, namely phytonutriceuticals (fibers, vitamins, minerals, and micronutrients such as polyphenols or carotenoids), have many benefits on health [9,10,11,12,13,14].

Molecules such as polyphenols or vitamins seem to alleviate negative impacts [15,16,17] as scavengers of free radicals [18] or reactive nitrogen species [19], and as potential inhibitors of carcinogenic substances formation in the stomach (notably flavanols against N-nitrosodimethylamine) [19]. The place of natural substances, such as polyphenols or vitamins in food products, is therefore gaining prominence and the understanding of those molecules’ roles in reformulated processed meats is thus paramount to enhance their nutritional quality [15,20].

Yet, too few data concerning the precise characterization of phytonutriceuticals’ chemical reactivity in processed meats are available for the manufacturers. There is a real need to deepen the mechanisms allowing vegetable molecules to modulate the detrimental health effects associated with their consumption. The mitigation of those negative impacts can be studied through the action of polyphenols and vitamins on two principal reactions involving the added sodium nitrite, being N-nitrosation and lipid oxidation, both mainly occurring in the digestive tract, and supposed to be spearhead of health issues after processed meat consumption. The catalysis of those reactions during digestion may be explained by the pro-oxidant environment of the gastrointestinal tract (low gastric pH, high oxygen pressure, body temperature) and the liberation of N-nitrosation and lipid oxidation, i.e., the most involved compounds (secondary amines and fatty acids, respectively) [4,21]. The need to screen the potential modulation of N-nitrosation and lipid oxidation induced by vegetable molecules during processed meats digestion in a model is therefore a challenge to tackle.

Some *in vitro* models have been designed to study the impacts of vegetable molecules on the chemical reactivity of those reactions’ end products. Several papers studied the mechanisms involving polyphenols and vitamins on N-nitrosation [22,23,24,25], but without a digestive environment, nor in relation with a particular product matrix. N-nitrosation in the oral cavity has been studied by working on models of acidified saliva [24,25,26,27]. Yet, focusing on the gastric compartment environment would be of major interest as it seems to promote N-nitrosation and lipid oxidation [16,28,29].

Indeed, *in vitro* digestive models have been designed to study lipid oxidation [29] and its inhibition by polyphenols [30,31]. The core of their model was an oil in water emulsion, characterizing the composition and the chemical state of lipids in the tract [32]. Lipids present in the emulsion not only allow to represent the complexity of a food bolus content, but also to model the chemical reactivity of N-nitrosation and lipid oxidation in an emulsified complex system. Nevertheless, to focus on a product of interest in a model is of major importance considering the current strategy of processed meats reformulation with plant molecules [7,33]. Hence, to the current knowledge, the specific study of different chemical reactions of processed meats leading to a possible digestive toxicity using *in vitro* models is scarce.

The aim of this paper was therefore to develop a method allowing to screen several polyphenols and vitamins for their specific action during digestion in a model depicting processed meat products. To reach that goal, the method must be rapidly efficient, reliable, and reproducible, which is a greater stake when working with emulsions. The stability of the emulsionized *in vitro* model will constitute one step of the present study. Then, an estimation of the reactivity of polyphenols and vitamins on those two key chemical reactions is expected in a processed meat model.

Two vitamins and four polyphenols were selected for the study. Ascorbic acid (vitamin C) and trolox (hydrosoluble form of vitamin E) were used as highly reactive and used vitamins in processed meats formulations. Then, four molecules of polyphenols of different classes were chosen, in order to potentially observe an impact of the molecule’s structure on the modulation of the chemical reactivity (chlorogenic acid for phenolic acids, rutin for flavonoids, and naringin and naringenin for an aglycone and a glycone form of polyphenol from the flavanones). As highly used in processed meats formulations, the combined effect of the selected plant molecules with ascorbic acid will be evaluated. Working with an emulsified medium may also affect the chemical reactivity of the molecules [34], which is why the modulation will also be assessed in two different medium: a simple aqueous medium and a complex emulsified one. Studying the combined effect of the vegetable molecules of interest with ascorbic acid and the impact of the model medium would therefore provide deeper insight on the modulation of N-nitrosation and lipid oxidation in a digestive environment of processed meat.

## 2. Materials and Methods

All the reagents, i.e., acetone, L-α-phosphatidylcholine (CAS: 8002-43-5; ≥50%), citric acid, sodium hydrogen phosphate, sodium nitrite (CAS: 7632-00-0; ≥99%), (+)-sodium L-ascorbate (CAS: 134-03-2; ≥98%), iron (II) sulfate heptahydrate (CAS: 7782-63-0; ≥99%), ethanol, N-acetyl-DL-tryptophan (CAS: 87-32-1; ≥98%), chlorogenic acid (CAS: 327-97-9; ≥95%), rutin hydrate (CAS: 207671-50-9; ≥94%), naringenin (CAS: 67604-48-2; ≥95%), (±)-naringin (CAS: 10236-47-2; ≥95%) and Trolox ((±)-6-Hydroxy-2,5,7,8-tetramethylchromane-2-carobxylic acid) (CAS: 53188-071) were purchased from Sigma-Aldrich (Saint Louis, MI, USA). Filter papers (Ref: 074121; Dim: 70 mm) were from Dutscher (Brumath, France). Sunflower oil (11 g of saturated fatty acids/100 g of oil) was from a local market (Auchan), and its amount of α-tocopherol was titrated by high-pressure liquid chromatography (HPLC) to 75.60 mg/100 g.

### 2.1. Oil in Water Emulsion

**Figure 1:**
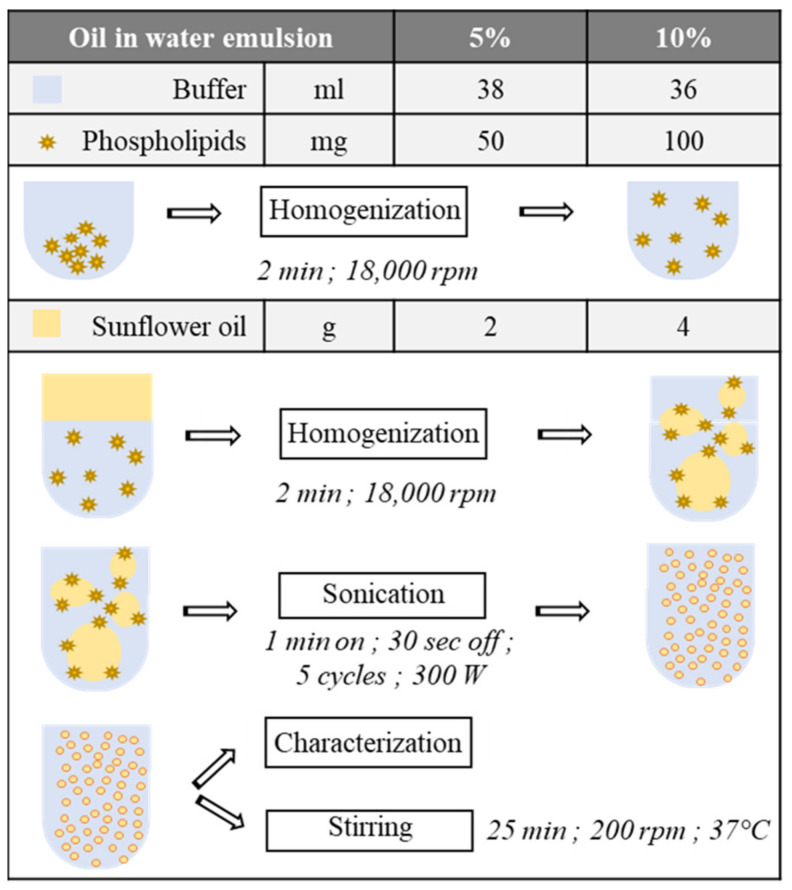
Fabrication process of the emulsified screening medium, with 5% or 10% of oil in water.

**Emulsion fabrication process.** The oil in water ultrasonic emulsion was stabilized with egg yolk phospholipids (L-α-phosphatidylcholine). Two proportions of lipids were tested: either a 5% or a 10% oil in water emulsion (stabilized with 0.4% or 0.8% of lecithins, respectively) (Figure 1). The aqueous phase was an acidic buffer solution (20 mM citric acid, 40 mM sodium hydrogen phosphate) adjusted to the pH of study, e.g., 2 or 3.5. The lipidic phase was made of crude sunflower oil. Emulsions were prepared in several steps (Figure 1). After adding the phospholipids in the buffer, a first homogenization was performed using a rotor–stator (Polytron PT 2500 E; Kinematica AG, Malters, Switzerland) at 18,000 rpm for 2 min at room temperature, in order to correctly scatter phospholipids aggregates in the buffer. Then, 2 or 4 g of sunflower oil was added in the solution. A second homogenization using the same parameters was performed on the solution before the emulsion sonication. The latter was conducted on melted ice with a Vibra Cell (VC-300; Thermo Fischer Scientific, Illkirch, France) sonicator, to prevent heating by sonication, for seven minutes: five cycles of 60 s active phase and 30 s off, at 300 W of power. The emulsion was directly put under stirring (200 rpm) in a preheated chamber (NB-205L; N-Biotek, Gyeonggi-Do, Korea) at 37 °C for 25 min after its fabrication.

**Emulsion granulometry analyses.** Once sonicated, the emulsion was directly characterized with a flow particle image analyzer (FPIA) (FPIA-3000; Sysmex, Kobe, Japan) in triplicate as a quality control. The analysis methodology was described by Promeyrat et al. in 2010 [35]. The sample analysis was preceded by two tests. The first was an auto-focus calibration with a commercial solution of latex spheres of 3.2 µm of diameter (Latex Microsphere Suspensions; Duke Scientific Corporation, Fremont, CA, USA), and 100 µL of the latter solution diluted in pure water (1/7; *v*/*v*) was injected for the analysis. The second was an automated background check review, counting the remaining particles in the FPIA. It was repeated until at the most 30 particles were present in the unit. The high-power field (HPF) selection mode was elected, allowing to analyze particles ranging between 1 and 40 µm of diameter with a 20-times magnification. Each measurement was automatically diluted in an electrolytic sheath buffer (7.1 g/L NaCl, 0.8 g/L surfactant, 2.0 g/L Tris, 0.2 g/L EDTA). Then, 100 µL of diluted sample in buffer (1/50; *v*/*v*) was injected in the chamber for its characterization.

Three criterions were selected for the characterization. First, the size of the particles was examined with the perimeter equivalent diameter (PED), in µm. Then, the shape was assessed with the circularity index, in percentage. Third, the density was measured and provided with the percentage of analyzed particles belonging to the set-up standards. The latest corresponded to the acceptable range for lipid droplets in an ultrasonic emulsion, meaning a PED lower than 10 µm [36] and a circularity belonging to the interval 90–100% [37].

### 2.2. Screening Model

**Figure 2:**
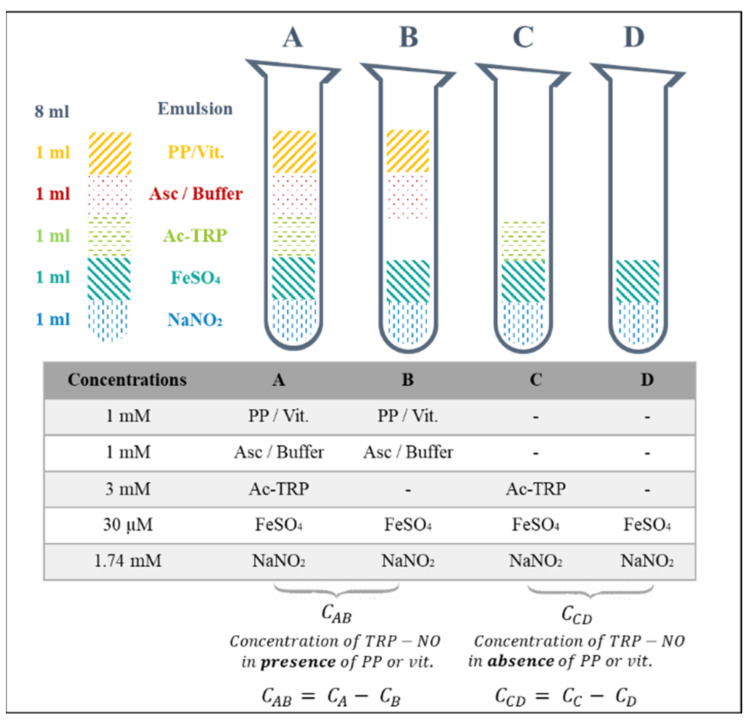
Experimental design to screen the modulation of the chemical reactivity induced by vegetable molecules. Asc: ascorbate; PP: polyphenol; Vit.: vitamin; FeSO_4_: iron (II) sulfate heptahydrate; NaNO_2_: sodium nitrite; Ac-TRP: acetyl-tryptophan; TRP-NO: N-acetyl-DL-nitroso-tryptophan, C: concentration.

**Screening model experimental design description.** The methodology designed to assess the action of polyphenols and vitamins on N-nitrosation and lipid oxidation is based on differential UV-visible spectrophotometry. For each pure molecule, four systems were created. The two firsts, A and B, were supported by reactions in the presence of the vegetable molecule; while C and D did not contain any of them. B and D were the respective blank for A and C against the polyphenol or vitamin. Different reagents were then introduced in the emulsion according to the reaction intended (Figure 2). The end products of N-nitrosation and lipid oxidation reactions were measured after 15 min of incubation in the steam room. A quantification of N-acetyl-nitroso-tryptophan and aldehydes was then obtained for each one of the four systems. Yet, the values resulting from B and D measurements corresponded to the spectral noise. Therefore, the amount of nitrosamines and aldehydes in the presence of polyphenol or vitamin, or not, was given by the subtraction of the different concentrations in each system, by considering the residual turbidity of the sample (Figure 2).

The methodology was used to assess three chemical reactivities: (1) the effect of polyphenols and vitamins in an emulsified model; (2) the combined effect of polyphenols and vitamins with ascorbic acid in an emulsified model, to explore a potential interaction with the compounds and ascorbic acid; and (3) the effect of polyphenols and vitamins in a simple aqueous buffer (without lipids), to explore the effect of lipids presence on the chemical reactivity.

**Screening *in vitro* model composition.** A cooked meat model was chosen as the referenced processed meat matrix, for its highly studied chemistry and known composition [38]. The main compounds involved in the reactions and their concentrations in those products were identified to design a comprehensive model of phytonutriceuticals’ chemical reactivity screening. Sodium nitrite is usually introduced up to 120 ppm with 300 ppm of ascorbate in France [39]. Nitrosamines’ synthesis depends notably on the presence of free iron [40] and is enhanced with acetyl-tryptophan [41]. Their respective amounts were set according to previous work at 30 µM [38] and 3 mM [40].

The volume of each system was similar and fixed at 13 mL: 5 mL of reactive compounds was added to 8 mL of emulsion, accordingly to the system, as described in the Figure 2. NaNO_2_ and FeSO_4_, at respective final concentrations of 1.74 mM and 30 µM, were systematically diluted in the buffer solution and introduced in every system. Then, according to the latter, different solutions were added. Acetyl-tryptophan (Ac-TRP) (3 mM) was prepared as stock solutions in pure ethanol, i.e., 120-fold stock, with 325 µL of this solution added in the system. N-acetyl-DL-tryptophan was selected according to Nakai et al. [41] at 3 mM in order to target the exclusive generation of the N-nitroso-tryptophan nitrosamine in the system (and not several species such as nitroso and/or nitro-tryptophan).

Chlorogenic acid, rutin, naringin, naringenin, and trolox, at a final concentration of 1 mM [42] and a volume of 325 µL, were prepared in either pure ethanol as well (system B), or in the ethanolic Ac-TRP solution (system A). When 325 µL of ethanolic solution (of either Ac-TRP or polyphenol/vitamin) was added, 675 µL of buffer was introduced to complete the requirement of 1 mL of addition. Ascorbate was solubilized in the buffer at 1 mM. All these solutions were aliquoted and stored at −80 °C.

### 2.3. Biochemical Assays

**Nitroso-tryptophan assay.** The specific absorbance of the nitrosamine bond was measured at 760 nm and 335 nm with a spectrometer (Jasco V770; Lisses, France), according to the method described by Nakai et al. in 1978 [41]. After 15 min of incubation, 500 µL of the sample was directly added to 2 mL of acetone and 150 µL of pure water for the nitroso-tryptophan concentration measurement. The solution was filtered after 2 min of vortex. The blank was composed of 2 mL of acetone and 650 µL of water. The absorption coefficient is 6100 M^−1^cm^−1^ [43]. Nitroso-tryptophan concentrations are expressed in µM.

**TBARS assay.** Lipid oxidation was assessed thanks to the thiobarbituric acid reactive substances (TBARS) method, enabling the quantitative measuring of the aldehyde function of molecules. The major one stemming from the oxidation chain reaction is the toxic malondialdehyde (MDA) [44]. After the incubation of the system, 300 µL of sample was frozen at −80 °C for the TBARS assay. The sample was added to 150 µL of thrichloroacetic acid (TCA) and 150 µL of thiobarbituric acid (TBA). The mixture was boiled for 10 min. The samples were then extracted with 1.2 mL of butanol during 15 min of centrifugation (4000 rpm, 4 °C) (SL 40R centrifuge; Thermo Scientific; Waltham, MA, USA). Organic phase absorbance was measured at 535 nm and 760 nm by spectrophotometry (Jasco V770; Lisses, France). The blank was made of butanol. Results are expressed in µmol of MDA equivalent per mg of lipids.

### 2.4. Statistical Analysis

Figures are reported as mean ± standard deviation of 4 independent measurements, for each concentration of every systems studied (A, B, C and D). Variance analyses (ANOVA) were realized with the software Statistica (V13.5; Statsoft Inc. Tulsa, OK, USA), with a Tukey post-hoc test (significance assessed by *p*-values < 0.05). The variables considered for respective modulation of N-nitrosation and lipid oxidation were the concentration of nitroso-tryptophan and the amount of TBARS. For the modulation of N-nitrosation, the factors were the polyphenols or vitamins, the presence of ascorbate, and the presence of emulsion or not. Lipid oxidation measurements were analyzed with the vegetable molecule, as in system A.

## 3. Results and Discussion

### 3.1. Optimization of the Heterogeneous Reactional Medium

The impact of pH and oil amount was studied on the characterization profiles of the emulsions in order to select the best parameters for the emulsion’s stability, and for the good relevance of the model. Two acidic pH were tested, 3.5 and 2, to symbolize the acidity of the beginning and mid digestion. Two percentages of oil amounts were investigated (5% and 10%) to assess the potential effect of lipids’ proportions on the emulsion profile. These oil percentages represented the level of lipids that can be found in a Western diet. Sunflower oil was selected for its highly oxidizable fatty acids composition, allowing to correctly assess a modulation of lipid oxidation reaction by polyphenols and vitamins by differential spectrophotometry.

**Figure 3:**
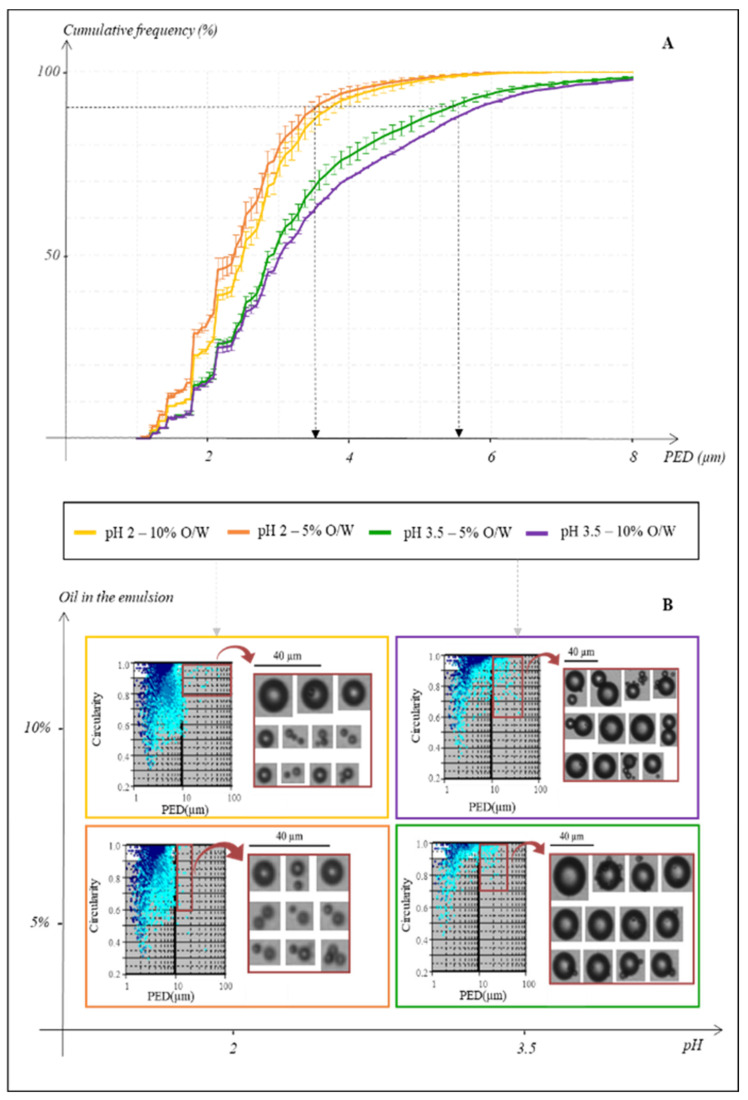
Characterization of emulsions according to the modulation of two parameters: pH (2 or 3.5) and the amount of oil in water (5% or 10%). (**A**): Cumulative frequencies of the droplets enumerated (%) as a function of the PED (µm). The differences of perimeters at 90% of the enumerated particles are highlighted. (**B**): Characterization profiles of the emulsions, representing the circularity of the particles as a function of the PED (µm). The blue colour scale indicates the density of the identified particles: from cyan blue for single droplets to dark blue for several of them. The white background corresponds to the most accurate particles eager to be lipid droplets (PED < 10 µm and circularity from 90% to 100%). Representative pictures shot by the FPIA of the largest particles of each emulsion are given.

Figure 3A depicts the distribution of the particle’s sizes in four different emulsions according to the cumulative frequencies, in percentage, as a function of the PED, in µm. Emulsions have an overall droplets PED lower than 10 µm. The differences induced by the pH modulation on droplets’ perimeters prevailed over the oil concentration ones. At pH 2, the heterogeneity of the reactional medium seemed to be reduced. For a given pH, no distinct difference between the emulsions was highlighted in relation to the oil amount. Indeed, at pH 2, almost 90% of lipid droplets (from both the 5% and 10% O/W emulsions) had a PED of 3.5 µm, when the size of 90% of the particles from pH 3.5 emulsions reached approximately a PED of 5.5 µm.

The profiles given by the FPIA represent the PED of the particles (µm) as a function of their circularity, with an index of the counted particles represented by a blue colour scale (Figure 3B). The prominent modification observable on the profiles was the pH impact, where the increase in the latter from 2 to 3.5 induced mainly changes in the distribution of the droplets in terms of PED and density (Figure 3A,B). For the same amount of oil, lipid droplets density seemed to be reduced with the rise in the pH value, leading to the formation of bigger oil agglomerates in the emulsions. Hence, the pictures shot by the FPIA showed that the largest particles of the emulsions, which are the most likely ones to cause destabilization, are almost three times larger at pH 3.5 than at pH 2. This observation was also effective with 10% of oil, although a bit less pronounced. Nonetheless, the fusion of several droplets was concrete at pH 3.5 as pictures have been shot of the fusion of lipid particles. The rise in the oil percent had less impact on the characterization profiles, although the density seemed to be risen as well as the number of large particles eager to destabilize the emulsion. This was particularly true for the pH 3.5 with 10% O/W emulsion.

Nevertheless, as illustrated by the pictures shot, the equipment may not distinguish very well close particles and therefore count them as entities (Figure 3B). In order to overcome this bias and to exclude possible air bubbles, agglomerates, and sample noise [37], the interval of circularity and perimeter analysis was narrowed. Thus, it was possible to select exclusively the droplets most subjects to correspond to lipidic particles thanks to the shape (90–100% of circularity) and the size (maximum size range of 10 µm of PED [36]) data, creating a window of study (blank background of the profile against the grey one). The software returned an approximate proportion of 80 to 85% of the enumerated particles belonging to this window.

The aim of this preliminary work was to set the correct parameters to optimize the emulsion. Indeed, as an emulsion is defined by its kinetics of destabilization [45], reaching greater stability over time, a correct reproducibility of its internal structure, and avoiding destabilization processes, are major stakes of the present development. Yet, those phenomena are promoted by an uneven distribution of the droplets in terms of size and shape [46]. Therefore, the lower the pH, the thinner particles in the emulsion, thus leading to a pH buffer choice of 2. Working with smaller lipid droplets also allows to rise the exchange surface, which enhances the reactivity within the solution and therefore optimizes the sensitivity of the model. Furthermore, as the reproducibility of the model was paramount to ensure the following chemical reactivity screening, the amount of oil in water was set at 5% in order to minimize the part of large droplets potentially able to destabilize the emulsion. Once those parameters set, with a constant stirring of the emulsion in a preheated even at 37 °C ensuring the physico-chemical environment of a gastric digestion, the modulation of the chemical reactivity could be assessed based on processed meats chemistry.

### 3.2. Chemical Reactivity Modulation

Quite a few relevant compounds and an emulsified medium were used. The complexity created is thus the main asset of the model herein developed, as it could depict the most complex chemistry occurring during processed meats digestion.

The chemical reactivity modulated by polyphenols and vitamins on lipid oxidation and N-nitrosation has been assessed in different models by differential UV-visible spectrophotometry. Every modulation is given compared to the control model without pure vegetable molecule, thus meaning that it can predict the effect of the lone substance’s presence in the design (Table 1).

There is a significative polyphenol modulation on lipid oxidation (*p*-value < 0.001) (Table 1). In the model without added ascorbate, chlorogenic acid, rutin, naringenin, and naringin significantly decreased lipid oxidation. This decrease was more pronounced for chlorogenic acid and rutin (about 88% fall) than for the flavanones (naringenin and naringin) (around 50% decrease). The same tendency with the same values was observed in the model with ascorbate, except for naringin. In contrast, for both models with and without ascorbate, vitamins had no effect on lipid oxidation. Hence, no effect attributed to the addition of 1 mM ascorbate with the other pure vegetable molecules (at 1 mM as well) was observed on lipid oxidation in the present design. Indeed, the rate of MDA equivalent was tantamount with or without the added ascorbate (Table 1A).

The assessment of lipid oxidation in a modelized gastric compartment is paramount as it was demonstrated to be a bioreactor enhancing this reaction [28]. The significant decrease in lipid oxidation induced by the previously cited molecules is consistent with the known chemical reactivity of these molecules. Chlorogenic acid, rutin, naringenin, and naringin display different structures as they come from different classes of phenolics (namely phenolic acids, flavonols, and flavanones, respectively), thus meaning that a a comprehensive overview of the representatives molecules’ chemical reactivity could eventually be obtained [4]. The modulation of lipid oxidation observed therefore upholds the good functionality of the present developed experimental design. The absence of ascorbate effect on lipid oxidation may be surprising, but this result has to be taken in the overall context of the model, with its complexity arising from the emulsified medium and, therefore, the differences in compounds’ solubilities, the concentrations of the different reagents, and the physico-chemical environment implemented.

Concerning N-nitrosation, the addition of rutin promoted the reaction in both emulsified models with and without added ascorbate, up to 36% and 50%, respectively. The same result was observed in the aqueous model (approximate increase of 36%). Whatever the model studied, no pure polyphenols nor vitamins could be discriminated as enhancers or inhibitors. This fact may arise from the lack of sensibility of the spectrophotometric method against the measured nitroso molecules. Working by differential spectrophotometry is a strength to complexify the model but, in the present case of less than 10% of synthetized nitroso-tryptophan (the maximum amount managed to be reached), this method might be limited to distinguish small modulations induced by the vegetable molecules. Yet, the system was efficient to highlight the effects on N-nitrosation.

Indeed, the impacts of structure and composition of the medium on N-nitrosation was studied by comparing the results obtained on the emulsionized model with those on the buffer model. Results showed a significant effect on N-nitrosation modulation (*p*-value < 0.001) (Table 1B). N-nitrosation was significantly reduced (by almost 30%) in the emulsified model, compared to the model containing only aqueous buffer.

Those results are consistent with previously shown mechanisms. Combet et al. demonstrated in 2010 that some phenolics modulated differently N-nitrosation according to the presence of lipids (under the form of bulk oil) or not [34]. They notably showed the enhancement of N_2_O_3_ synthesis in a lipidic compartment (up to 400 times) [34,47]. This highly reactive species is able to react with both secondary amines leading to nitrosamines [34,40,47] or with lipidic peroxyl radicals, leading to nitroso-peroxyl radicals [48,49], according to chemical competitions between the different nitrite targets. In absence of lipids, the nitroso-peroxyl radicals’ synthesis path does not exist and the balance may therefore lean towards a promoted N-nitrosation reaction compared to the measurements in the emulsified medium. Yet, to the current knowledge, it is the first time that N-nitrosation is assessed in an oil in water emulsion, which is the most representative medium to model *in vitro* digestion of food bolus. Those results not only confirm the present developed model to study chemical reactivity modulations, but also deepen a rarely explored aspect explored, i.e., the reactivity of N-nitrosation in a complex environment close to the digestive one, herein represented by the use of an oil in water emulsion.

Moreover, a slight but significant reduction in N-nitrosation was noted under the combined presence of added ascorbate to the polyphenol or vitamin in the emulsified medium (*p*-value < 0.05), which was around a 12% decrease (Table 1C). Nonetheless, the absence of a distinguished modulation by ascorbate in present systems with or without the different pure vegetable substances may be surprising as ascorbate has been demonstrated several times to inhibit N-nitrosation alone [9,40,50] or with other phytomicronutrients [22]. To the current knowledge, it is the first time that the chemical reactivity of N-nitrosation is assessed in an emulsified medium. Yet, the chemical reactivity of ascorbate in presence of a multiphase system may be more complex than in an aqueous system, as it was shown that N-nitrosation modulation may vary from inhibition to promotion according to the presence of bulk lipids or not [47]. Moreover, ascorbate may also modulate the stability of other polyphenols when introduced jointly [51], which emphasizes the complexity of the chemical interactions between the different compounds. This effect result is interesting within the framework of processed meats reformulation, where ascorbate is very often added. The model was herein efficient to identify a modulation of N-nitrosation.

## 4. Conclusions

The main purpose of this model was to characterize the existence of modulations induced by polyphenols and vitamins on targeted chemical reactions. It was conceived to help screening several vegetable molecules in good time and to obtain a global idea of their impact in a representative medium of processed meat products. It was shown to be efficient, and was authorized to demonstrate variations induced by polyphenols and/or vitamins, the medium structure, and the medium composition. The oil in water emulsion model was optimized to be reproducible, stable over time, and practical to implement in the context of a digestive physico-chemical environment. Moreover, the modulations herein shown have to be interpreted within the overall context of the study: the nature of the different substances, the emulsionized or non- emulsionized structure of the medium, and the method by differential UV-visible spectrophotometry. The present work provides information to wisely reformulate processed meats to enhance their nutritional qualities. Thanks to the present characterized modulations, deeper analyses with more sensitive methods would bring out the mechanisms underlying the modulations highlighted, as well as the involved and the neo-formed molecules at stake in the reactions.

## Figures and Tables

**Table 1:** Polyphenols and vitamins modulation on lipid oxidation and N-nitrosation screening. The control consists in no antioxidant. Each result is given as mean ± SD of 4 independent measurements. Significate differences are given relatively to the control. (*: *p*-value < 0.05; ***: *p*-value < 0.001; NS: *p*-value > 0.05).

	**Lipid Oxidation** **(nmol MDA Equivalent/mg Lipids)**		**N-Nitrosation (%)**		
	**Emulsion**	**Emulsion**	**Buffer**	**Emulsion**	**Emulsion**
	-	Ascorbate	-	-	Ascorbate
**Control**	**0.462 ± 0.052**	**0.367 ± 0.044**	**8.148 ± 1.601**	**5.825 ± 0.983**	**5.096 ± 1.044**
Ascorbate	0.367 ± 0.044 ^NS^	0.592 ± 0.068 ^NS^	6.857 ± 2.653 ^NS^	5.096 ± 1.044 ^NS^	4.245 ± 1.045 ^NS^
Trolox	0.397 ± 0.051 ^NS^	0.454 ± 0.077 ^NS^	7.384 ± 1.283 ^NS^	4.354 ± 0.327 ^NS^	4.687 ± 2.096 ^NS^
Chlorogenic Acid	0.057 ± 0.013 ^***^	0.058 ± 0.020 ^***^	5.500 ± 2.864 ^NS^	6.630 ± 3.310 ^NS^	4.337 ± 1.564 ^NS^
Rutin	0.052 ± 0.008 ^***^	0.035 ± 0.007 ^***^	12.743 ± 1.940 ^***^	9.112 ± 1.317 ^***^	10.458 ± 0.893 ^***^
Naringenin	0.227 ± 0.030 ^***^	0.196 ± 0.032 ^***^	7.485 ± 0.917 ^NS^	6.215 ± 2.176 ^NS^	6.026 ± 0.417 ^NS^
Naringin	0.287 ± 0.017 ^***^	0.407 ± 0.013 ^NS^	8.351 ± 0.592 ^NS^	6.618 ± 1.398 ^NS^	5.053 ± 1.376 ^NS^
	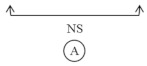		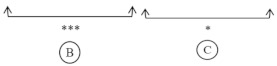		

A: Effect of the ascorbate presence on lipid oxidation. B: Effect induced by the medium (emulsified or aqueous) on N-nitrosation. C: Effect of ascorbate addition on N-nitrosation in the emulsified medium.
